# Editorial: Application of PKPD modeling in drug discovery and development

**DOI:** 10.3389/fphar.2025.1614689

**Published:** 2025-06-26

**Authors:** Hong-Can Ren, Xiao Zhu, Kun Hao, Si-Yuan Le

**Affiliations:** ^1^ Department of Drug Metabolism and Toxicity, Shanghai Pharmaceuticals Holding Co Ltd, China; ^2^ Department of Clinical Pharmacy and Pharmacy Administration, School of Pharmacy, Fudan University, China; ^3^ Department of Drug Metabolism, China Pharmaceutical University, China; ^4^ Department of Translation Medicine, Genfleet Therapeutics (Shanghai) Inc, China

**Keywords:** drug discovery and development, PKPD, PBPK, molecular design, model-informed drug development (MIDD), special populations, personalized medicine

## Introduction

In the rapidly evolving field of pharmaceutical research, PKPD modeling has emerged as a cornerstone of modern drug development. By integrating pharmacokinetics (how the body processes drugs) and pharmacodynamics (how drugs affect the body), PKPD modeling provides a systematic framework for understanding the complex interplay between drug efficacy and safety. Pharmacokinetics can be modeled using compartmental models or physiologically based pharmacokinetic (PBPK) models, which offer mechanistic insights into drug absorption, distribution, metabolism, and excretion (ADME) processes (Ren et al., 2019; Ren et al., 2022). These models range from empirical and semi-mechanistic approaches to highly complex frameworks incorporating anatomical and physiological data.

The integration of PKPD modeling into the drug development pipeline is not merely a technical advancement but a strategic necessity. It enables informed decision-making at every stage, optimization from molecular design to clinical trial (Chen et al., 2024; Liao et al., 2024; Liu et al., 2024; Wanika et al., 2024). Recent advancements in oncology highlight its transformative potential, where PKPD models predict chemotherapy efficacy, unravel drug resistance mechanisms, and accelerate the translation of research into clinical practice. Beyond chemotherapy, PKPD modeling has broad applications in developing novel therapeutic agents like antibody-drug conjugates, underscoring its versatility in addressing diverse therapeutic challenges.

As diseases grow more complex and the demand for personalized medicine intensifies, PKPD modeling becomes indispensable. Coupled with spatial and temporal tumor modeling, it allows researchers to explore drug dynamics comprehensively, leading to therapies that are both effective and tailored to individual patient needs. PBPK models, in particular, provide a mechanistic understanding of drug behavior across species and routes of administration, reducing reliance on animal testing, enhancing translational success, and drug interaction prediction (Jiang et al., 2023; Liao et al., 2024; Ren et al., 2021; Yang et al., 2025). This editorial explores how PKPD modeling is reshaping drug discovery and development, providing a robust framework for understanding mechanism of actions and predicting patient outcomes.

### Optimizing molecular design through PKPD insights

PKPD modeling is revolutionizing molecular design by enabling data-driven decisions. The study by Sanchez et al. on bispecific antibodies exemplifies how mathematical models can predict the impact of binding affinity on trimeric complex formation and patient outcomes *Combining mathematical modeling, in vitro data and clinical target expression to support bispecific antibody binding affinity selection: a case example with FAP-4-1BBL* Sanchez et al. By simulating FAP-4-1BBL’s binding dynamics, researchers identified optimal affinity thresholds that maximize therapeutic benefits across diverse patient populations. This approach contrasts sharply with traditional methods, reducing reliance on trial-and-error and accelerating the identification of promising candidates.

Similarly, PBPK models for protein therapeutics in rabbits offer a predictive framework for understanding species-specific differences and guiding preclinical studies *Physiologically based pharmacokinetic models for systemic disposition of protein therapeutics in rabbits* Jairam et al. These models, which incorporate physiological parameters and target interactions, provide a foundation for translating findings across species and routes of administration, thereby de-risking early-stage development.

### Accelerating clinical development with model-informed strategies

The COVID-19 pandemic underscored the need for rapid clinical development, a challenge addressed through model-informed drug development (MIDD). The leritrelvir study demonstrated how population pharmacokinetic analyses could optimize dosing regimens and trial designs within compressed timelines *Model informed dose regimen optimizing in development of leritrelvir for the treatment of mild or moderate COVID-19* Wang et al. By leveraging existing data and predictive modeling, researchers efficiently navigated regulatory requirements and clinical complexities, highlighting PKPD modeling’s role in public health responses.

Recent advances in PBPK modeling have also been instrumental in optimizing first-in-human dose predictions, as demonstrated in studies involving efalizumab, a therapeutic protein used in psoriasis treatment *PBPK-based translation from preclinical species to humans for the full-size IgG therapeutic efalizumab*. Franz et al. developed PBPK models for efalizumab across three species (rabbit, non-human primate, and human), incorporating parameters related to FcRn binding and target-mediated drug disposition (TMDD). Their study revealed that while FcRn affinity parameters cannot be directly translated between species, TMDD-related parameters can be reliably translated from non-human primates to humans. This finding underscores the potential of PBPK modeling to reduce reliance on animal testing and enhance the precision of human dose predictions.

### Addressing unmet needs in special populations

PKPD modeling is particularly valuable in addressing therapeutic challenges in special populations. The investigation into cetirizine’s off-label use in pediatrics reveals how modeling can rationalize dose escalation, ensuring safety and efficacy in children *Model-based exploration of the rationality of off-label use of cetirizine in Chinese pediatric patients: a prospective cohort study* Liu et al. By simulating exposure across different ages and doses, researchers provided evidence-based guidance for clinical practice, filling gaps left by insufficient premarket data.

### Expanding the frontiers of personalized medicine

PKPD modeling is pushing the boundaries of personalized medicine. The bispecific antibody study’s analysis of FAP expression heterogeneity illustrates how modeling can predict patient-specific responses, moving away from one-size-fits-all approaches. This capability is echoed in the protein therapeutics study, where species-specific PBPK models pave the way for tailored preclinical strategies, enhancing translational success rates *Population pharmacokinetics and individualized dosing of tigecycline for critically ill patients: a prospective study with intensive sampling* Su et al.

Beyond oncology and pediatrics, PKPD modeling is also transforming treatment strategies in other therapeutic areas, such as inflammatory bowel disease (IBD). A recent study demonstrated the value of PKPD modeling in optimizing infliximab induction dosing to achieve clinical remission in patients with Crohn’s disease *Optimising infliximab induction dosing to achieve clinical remission in Chinese patients with Crohn’s disease* Zhu et al. By leveraging patient-specific data and modeling insights, researchers were able to identify optimal dosing regimens that maximize therapeutic benefits while minimizing adverse effects.

## Conclusion

PKPD modeling is not merely a technical tool but a transformative paradigm in drug development. It bridges preclinical and clinical research, offering unprecedented insights into drug behavior and patient outcomes. The studies highlighted herein demonstrate its potential to optimize molecular design, accelerate clinical trials, and enhance therapeutic precision. As we embrace this modeling-driven approach, the future of drug development promises to be more efficient, patient-centered, and innovative, ultimately delivering greater value to patients and society alike.
